# Analyzing temperature, humidity, and precipitation trends in six regions of Thailand using innovative trend analysis

**DOI:** 10.1038/s41598-024-57980-5

**Published:** 2024-04-02

**Authors:** Wissanupong Kliengchuay, Rachaneekorn Mingkhwan, Nuttapohn Kiangkoo, San Suwanmanee, Narut Sahanavin, Jira Kongpran, Htoo Wai Aung, Kraichat Tantrakarnapa

**Affiliations:** 1 Faculty of Tropical Medicine, Mahidol University, Krung Thep Maha Nakhon, Thailand; 2 Environment, Health and Social Impact Unit, Faculty of Tropical Medicine, Mahidol University, Krung Thep Maha Nakhon, Thailand; 3 Faculty of Public Health, Mahidol University, Krung Thep Maha Nakhon, Thailand; 4 Faculty of Physical Education, Srinakharinwirot University, Na-Khonnayok, Thailand; 5 School of Public Health, Walailak University, Nakorn Srithammarat, Thailand

**Keywords:** Sen’s slope estimator, Humidex, Temperature change, Mann–Kendall test, Innovative trend analysis (ITA), Climate sciences, Environmental sciences

## Abstract

The change of temperature and weather parameters is a major concern affecting sustainable development and impacting various sectors, such as agriculture, tourism, and industry. Changing weather patterns and their impact on water resources are important climatic factors that society is facing. In Thailand, climatological features such as ambient temperature, relative humidity, and precipitation play a substantial role in affecting extreme weather events, which cause damage to the economy, agriculture, tourism, and livelihood of people. To investigate recent serious changes in annual trends of temperature, relative humidity, and precipitation in Thailand, this study used the Mann–Kendall (MK) test and innovative trend analysis (ITA) methods. The MK test showed that all six regions had an upward trend in temperature and humidity index (humidex, how hot the weather feels to the average person), while relative humidity and precipitation showed both upward and downward trends across different regions. The ITA method further confirmed the upward trend in temperature and humidex and showed that most data points fell above the 1:1 line. However, the upward trend in most variables was not significant at the 5% level. The southern and eastern regions showed a significant upward trend in relative humidity and humidex at a 5% level of significance according to the MK test. The output of this study can help in the understanding of weather variations and predict future situations and can be used for adaptation strategies.

## Introduction

Climate change is a major natural concern in the twentieth century, with challenges to sustainable development and its impact^1^. Extreme weather events cause disruptions worldwide. Inclement weather has an almost fluctuating effect on ecosystems and natural resources everywhere, and some sectors, such as agriculture, tourism, and industry, are vulnerable in terms of a country’s development and human life. For examples, changes in climate could affect several parameters that influence the alteration of agricultural products. Consequently, this could have an impact on industries related to agriculture products. However, climate change also affects the tourism business; destination choices might change based on temperature and extreme weather events^2,3^. Changing weather patterns and their impact on water resources are important climatic features that pose a challenge to society^4,5^. Regarding the relationship with global warming, there are strong signs that rainfall and temperature changes are already taking place on a global scale^6^. In addition, IPCC reported that Climate Change has caused the effects to various sectors Including the consequences of human health impacts. The largest adverse impacts observed in many regions in Africa, Asia, Central and South America, least developed countries(LDCs), Small Islands and the Arctic^4^. The estimation of future climate variables change might increase the ambient temperature under the scenarios of IPCC and would cause the change of ecosystem^7–9^. Tropical countries are vulnerable to the change of weather pattern the and variability due to the complicated reactions among land, oceans, and the atmosphere^10^. Three major weather parameters patterns, the El Nino-Southern Oscillation (ENSO), Artic Oscillation (AO), and Indian Ocean Dipole (IOD), possibly influence climate phenomena in Asia and Thailand^11,12^. Additionally, atmospheric-ocean interactions in the tropical Pacific Ocean fluctuate periodically between cold (La Niña) and warm episodes (El Nino)^10^.

In Thailand, climatological features and the effects of local phenomena such as ambient temperature, relative humidity, and precipitation are the most important factors affecting extreme surface temperature, floods, drought and sea level rise. This situation has damaged the gross domestic product (GDP), agriculture, tourism and the livelihood of people. However, ambient temperature, relative humidity, and precipitation have been studied in the fields of climate science and hydrology and are frequently used to trace the extent and magnitude of the change of the weather and variability^13^. The Mann–Kendall (MK) test and the Theil-Sen approach (TSA) are the most useful methods to investigate the trend analysis of annual data on atmospheric and precipitation concentrations^1,14,15^. The Mann–Kendall (MK) test is unaffected by the actual distribution of the data, regardless of whether it is nonparametric. However, the limitation of the MK test is that it does not identify the high, medium or low category of trends. Thus, the application of innovative trend analysis (ITA) methods is useful, which is a graphic technique to explore trends from data and to avoid errors in detecting hidden significant trends^15^. The ITA method was recently developed by Sen^16^ and was used to analyze the trends of environmental, hydrological, and meteorological variables^17–20^. Trend analysis of climate factors were applied for many regions^21–26^.The global warming, rainfall pattern, temperature changes, as well as other parameter changing pattern will theoretically affect to the temperature, humidity and precipitation. Especially in Asian countries, the changing of weather, extreme weather event has affected the health of children and woman^27^. Previous studies found the changing of temperature will consequently effect the diseases transmission and the biological process will occur at a faster rate when the temperature is high^28^. Moreover, the high humidity correlated with a higher number of malaria cases and other infectious diseases as well as diarrhea^29^, while the precipitation change will also impact on the forest carbon storage which will play a major role in sequestering anthropogenic CO_2_^30^. Trend analysis is utilized to examine the direction of climate parameters, providing insights into the changing parameters that could be presented in annual and seasonal patterns. Moreover, this method can highlight regional differences, such as the distinct seasonal patterns observed in different regions of Thailand^31^.Thus, this study aims to analyze temperature, humidity, and precipitation trends in Thailand from 2001 to 2020 across six regions using the Mann–Kendall and ITA methods, with a focus on understanding climate change's impact. The output of this study was temperature, relative humidity and precipitation trends and their descriptive statistics, which can be used to understand weather variations and further predict the situation in the future.

## Methods

### Study area and data

**Figure 1:**
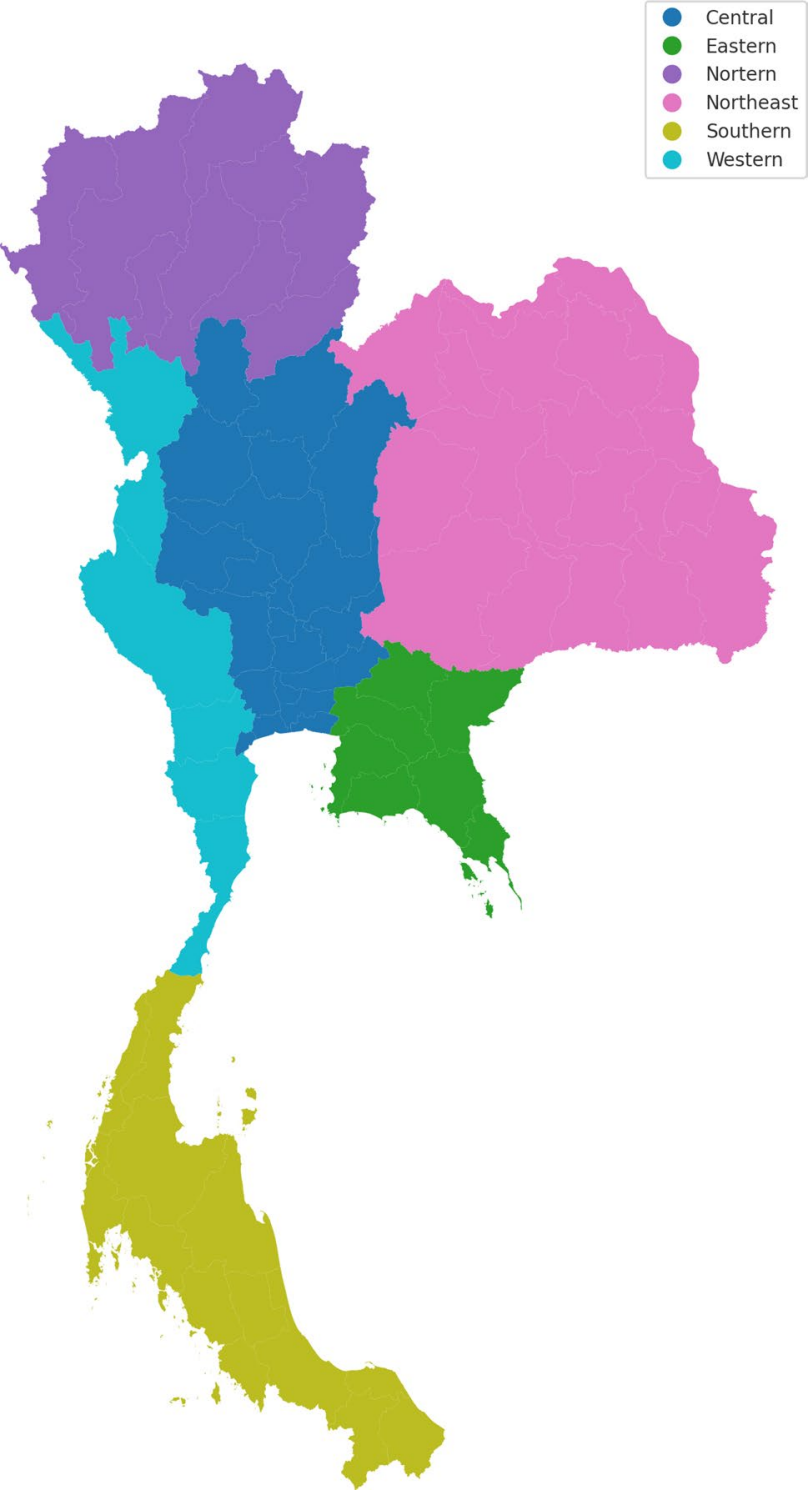
The classification of Thailand into six regions.

This study focused on 6 regions in Thailand (Fig. 1): the northern, southern, eastern, western, northeastern and central regions. The criteria for region classification are geography, culture including meteorological factors or climate factors. (Thai Meteorological Department, 2024). Trend analysis requires extended time-series data to show changes in climate patterns and accurately show ambient air temperature, relative humidity, precipitation and humidex trends. The annual data spanning from 2001 to 2020 were obtained from 127 stations: 17 stations in the north, 27 in the south, 15 in the east, 12 in the west, 31 in the northeast, and 25 in the central region, in a total of 77 provinces. Furthermore, in addition to monthly analysis, all data were accumulated annually to be analyzed for trends. Table 1 presents the characteristics and information of the six regions.

**Table 1:** The characteristics and details of each region.

No	Region	Number of Provinces (77)	Location (Decimal degrees)	Area (km^2^)	Population
1	Northern	9	19, 99	96,077	6,350,499
2	Southern	14	8.0592, 99.9756	73,848	9,454,193
3	Eastern	7	13.45, 101.6	34,481	4,841,806
4	Western	5	14, 99.5	53,769	3,430,314
5	Northeast	20	16, 103	167,718	22,017,248
6	Central	22	15, 100	91,798	20,183,134

### Data analysis

A statistical analysis of meteorological parameters such as ambient air temperature, relative humidity, and precipitation was conducted in this study using the following tools.

#### Climate classification

The classification of De Martonne, a numerical indicator of the degree of climate dryness at a given location and classify in relation to the water availability^32^ was used in this study to classify the climate regions of Thailand. This method has been used in many types of research^33^, as given in 7 types of aridity. These include aridity index values below 10 (I < 10), semiarid (I = 10 to 20), Mediterranean (I = 20 to 24), semihumid (I = 24 to 28), humid (I = 28 to 35), very humid (I = 35 to 55), and extremely humid (I > 55). The annual values of the DM aridity index, IaDM and ImDM can be represented by the following equations.

#### Humidex (HD)

The HD was first applied in Europe and the United States, where it was used by meteorological organizations to determine the likelihood of heat stress and thermal comfort conditions in a particular area and reported to the public^34,35^. The HD represents the warming effect perceived by people due to the lack of evaporation of body moisture, making them feel overwhelmed or strained, usually in summer^36^. The humidex will be used to calculated the relative humidity, the HD was calculated using Eq. 1^34,36^:

1 $$ {\text{Humidex}} = {\text{T }} + \, \left[ {{5}/{9 }* \, \left( {{\text{e}} - {1}0} \right)} \right] $$

where e is the vapor pressure [6.112 × (10^(7.5 * T/ (T + 237.7)^)] * (RH/100)), T is the ambient air temperature (°C) and RH is relative humidity (%). Heat produces a degree of personal stress that can vary according to the age and physical condition, health status, activity, etc., of each person. Dangerous ranges, humidex scales and symptoms are listed in Table 2.

**Table 2:** Interpretation of the humidex (HD) in terms of thermal comfort^36^.

DI range (°C)	Discomfort conditions	Symptoms
Less than 29 °C	No discomfort	Slight discomfort. Fatigue on prolonged exposure or physical activity
30–39 °C	Some discomfort	Discomfort increasing. Possible heat stroke or exhaustion produced by physical activity or simple exposure to open air
40–45 °C	Great discomfort or avoid exhaustion	Heatstroke on continued exposure or physical activity. Avoid activity. Search for a cooler area
Above 45 °C	Dangerous	Imminent heat stroke on continuous exposure

#### Mann–Kendall test and Sen’s slope estimator

The Mann–Kendall test in this study was used to analyze the changing trends of temperature, relative humidity, precipitation and the HD throughout the study period using RStudio version 2023.06.0 (Posit Software, Boston, MA) (i.e., the ‘trend’ package version 1.1.4). MAKESENS version 1.0 was used to prepare the graph of Sen’s slope estimate.

The Mann–Kendall test S statistic was calculated using the following formulas:

2 $$S= \sum_{k=1}^{n=1}\sum_{j=k+1}^{n}sgn({x}_{j}-{x}_{k})$$

where xj and xk are the annual values in years j and k, j > k, respectively, and

3 $$ sgn\left( {x_{j} - x_{k} } \right) = \left\{ {\begin{array}{*{20}c} 1 & {if} & {x_{j} - x_{k} > 0} \\ 0 & {if} & {x_{j} - x_{k} = 0} \\ { - 1} & {if} & {x_{j} - x_{k} < 0} \\ \end{array} } \right. $$

The variance of S was calculated by the following equation, which takes into account possible associations:

4 $$ {\text{In}}\;{\text{ n}} > {1}0\; {\text{VAR }}\left( {\text{S}} \right) \, = \frac{1}{18}\left[ {n\left( {n - 1} \right)\left( {2n + 5} \right) - \mathop \sum \limits_{p = 1}^{q} t_{p} \left( {t_{p} - 1} \right)(2t_{p} + 5} \right] $$

Here, q is the number of linked groups, and t_p_ is the number of data values in the p^th^ group. The values of S and VAR(S) are used to compute the test Z statistic as follows:

5 $$ \begin{aligned} {\text{In}}\;{\text{ S}} & > 0{\text{ Z}} = \frac{S - 1}{{\sqrt {Var\left( S \right)} }} \\ {\text{In}}\;{\text{ S}} & < 0{\text{ Z}} = { }\frac{S + 1}{{\sqrt {Var\left( S \right)} }} \\ {\text{In}}\;{\text{S}} & = 0{\text{ Z}} \\ \end{aligned} $$

The presence of a statistically significant trend was evaluated using the Z value. A positive (negative) value of Z indicates an upward (downward) trend. The Z statistic had a normal distribution. To test for either an upward or downward monotonic trend (a two-tailed test) at the α level of significance, H_0_ was rejected if the absolute value of Z was greater than Z_1−α/2_.

Sen’s slope method was used to estimate the true slope of an existing trend (as change per year) in this study. The slope (b) of a trend in sample data estimated by the Theil-Sen approach was as follows:

6 $${{\varvec{T}}}_{{\varvec{i}}}=\left(\frac{{x}_{t}-{x}_{{t}^{*}}}{t-{t}^{*}}\right)$$

where ${x}_{t}$ and ${x}_{{t}^{*}}$ are data values at time t and t*, respectively. In the equation above t > t*, the median of these n values of ${{\varvec{T}}}_{{\varvec{i}}}$ is represented by Sen’s slope of estimation (true slope) in Eq. 6.

7 $${Q}_{med}= {Q}_{(n+2)/2}$$

8 $${Q}_{med}= {({Q}_{(n)/2}+Q}_{(n+2)/2})/2$$

Sen’s estimator Q_med_ was calculated using the above equation depending upon whether n was odd or even. Then, Q_med_ was computed using a 100% (1 − α) confidence interval using a nonparametric test depending upon a normal distribution. A positive value of Q_i_ indicates an upward trend, while a negative value characterizes a downward trend of time series data^37^.

#### Innovative trend analysis

Innovative trend analysis (ITA) has been used for hydrometeorological observations^38–40^, and its accuracy has been compared with the results of the MK method^13,15^. This intuitive method is capable to identify the trends in different time series subcategories and can be applied regardless of distribution assumptions. In the ITA method, data points are divided into equal halves and then sorted in ascending order. After that, the two halves are placed on the Cartesian coordinate system, namely, *xi*,*i* = 1,2,…,*n*2 on the horizontal axis and *xj*,*j* = *n*2 + 1,*n*2 + 2,…,*n* on the vertical axis^15^.

### Ethics approval and consent to participate

This study was approved by the Ethics Committee of the Faculty of Tropical Medicine, Mahidol University TMEC 21-055, in compliance with the Declaration of Helsinki, ICH guidelines for Good Clinical Practice and other international guidelines for human research protection. Informed consent was not required for this study due to the use of secondary data.

## Results and discussion

**Figure 2:**
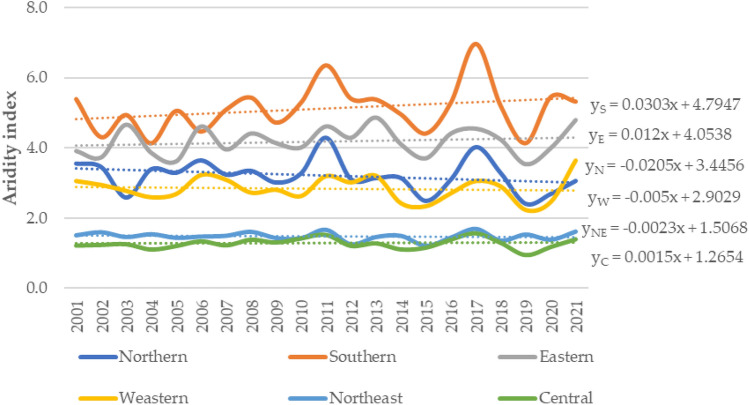
The distribution and trend of the mean annual De Martonne aridity index (I) by region from 2001 to 2020. The aridity trend is presented with 6 lines and a trend equation. The trend showed upward (drier direction) in Southern, Eastern, and Central region of Thailand, while the rest of regions demonstrate the downward trend (wetter direction).

As shown in Fig. 2, trends were positive in 3 regions and negative in 3 regions, which means that the climate was arid. The descriptive results of the distribution of the mean annual De Martonne aridity index classification and its trend for six regions between 2001 and 2020 are illustrated in Table 3. Table 4 presents the results of analyses of the climate sample by the Mann–Kendall test and Sen’s slope estimator for temperature, relative humidity, precipitation and humidity at each station during the study period.

**Table 3:** Descriptive statistics during the study period.

Region	Meteorological factor	Min	Max	Mean	SD	Median	1st Quartile	3rd Quartile	Skewness	Kurtosis
Northern	Climate classification	2.4	4.3	3.2	0.5	3.2	3.1	3.4	0.3	0.3
Southern		4.1	7.0	5.1	0.7	5.2	4.7	5.4	0.8	1.1
Eastern		3.5	4.9	4.2	0.4	4.1	3.9	4.5	0.1	−1.1
Western		2.2	3.6	2.8	0.3	2.8	2.6	3.1	0.2	−0.2
Northeast		1.2	1.7	1.5	0.1	1.5	1.4	1.5	−0.4	0.0
Central		1.0	1.6	1.3	1.0	1.3	1.2	1.4	0.0	0.1
Northern	Temperature (Degree Celsius)	25.1	26.6	25.9	0.4	25.8	25.7	26.1	−0.2	−0.1
Southern		27.2	29.4	27.6	0.5	27.5	27.5	27.7	3.1	10.1
Eastern		27.6	28.7	28.2	0.3	28.1	28.0	28.3	−0.2	−0.3
Western		26.2	27.5	27.0	0.3	27.1	26.9	27.3	−0.6	0.0
Northeast		26.1	27.9	27.0	0.4	27.0	26.9	27.3	−0.5	0.5
Central		27.6	28.9	28.3	0.4	28.3	28.1	28.6	−0.2	−0.4
Northern	Relative Humidity (%)	71.9	78.5	75.6	1.8	75.6	74.7	76.9	−0.5	−0.3
Southern		79.4	83.1	81.1	0.8	81.2	80.6	81.5	0.3	1.1
Eastern		74.2	79.3	77.0	1.1	77.0	76.5	77.5	−0.3	0.8
Western		72.0	77.6	75.1	1.3	75.1	74.2	76.0	−0.3	−0.2
Northeast		70.9	75.2	73.5	1.2	73.5	72.8	74.4	−0.4	−0.7
Central		71.2	75.3	73.6	1.1	73.7	72.9	74.4	−0.3	−0.6
Northern	Precipitation (%)	87.8	150.5	115.4	15.7	114.5	110.4	121.2	0.3	0.2
Southern		155.1	260.2	192.9	24.7	194.7	176.7	202.1	0.8	1.2
Eastern		136.5	184.8	159.7	14.8	156.3	148.8	173.2	0.1	−1.2
Western		83.7	134.0	105.4	12.1	104.0	98.7	113.0	0.2	−0.1
Northeast		100.2	144.2	123.7	10.7	121.7	119.1	127.5	0.0	0.0
Central		78.5	133.0	107.3	12.9	105.7	101.2	114.6	0.0	0.1
Northern	Humidex	33.0	35.1	34.3	0.6	34.4	34.0	34.7	−0.9	0.1
Southern		37.9	42.3	38.7	0.9	38.5	38.4	38.7	3.3	11.2
Eastern		37.8	39.8	38.9	0.6	38.9	38.4	39.3	−0.2	−1.0
Western		35.1	37.1	36.4	0.5	36.5	36.2	36.7	−1.1	0.7
Northeast		34.5	37.1	36.05	0.7	36.1	35.7	36.7	−0.6	0.0

**Table 4:** The results of the Mann–Kendall test and Sen’s slope estimator for temperature, relative humidity and precipitation at each station during the study period. The Sen’s slope estimates determined by the MK statistic (Z) had positive values of 1.58, 0.56, 0.98, 0.73, 0.97, and 1.80.

Meteorological parameter	Test	Mann–Kendall test (2001–2020)					
		N	S	E	W	NE	C
Temperature	Mann–Kendall test (z,s)	1.58, 53.0	0.56, 19.0	0.98, 33.0	0.73, 25.0	0.97, 33.0	1.80, 60.0
	Sen’s slope estimator	0.02	0.0	0.01	0.01	0.01	0.02
	Kendall’s tau	0.26	0.10	0.16	0.12	0.16	0.30
	Var(S)	1080.33	1026.33	1072.33	1081.67	1075.00	1076.00
	alpha	0.05	0.05	0.05	0.05	0.05	0.05
	p value	0.11	0.57	0.32	0.47	0.33	0.07
Relative humidity	Mann–Kendall test (z,s)	−1.06, −36.0	1.85,62.0	2.30, 77.0	−0.06, −3.0	0.39, 14.0	−0.09, −4.0
	Sen’s slope estimator (Ph. year)	−0.09	0.05	0.08	−0.003	0.02	0.0
	Kendall’s tau	−0.17	0.30	0.37	−0.01	0.07	−0.02
	Var(S)	1092.67	1090.00	1093.67	1093.67	1094.67	1090.00
	alpha	0.05	0.05	0.05	0.05	0.05	0.05
	p value	0.29	0.06	0.02	0.95	0.69	0.93
Precipitation	Mann–Kendall test (z,s)	−2.14, −72.0	1.21,41.0	0.815,28.0	0.57, −20.0	−0.79, −27.0	0.60,21.0
	Sen’s slope estimator (mm, year)	−0.83	0.99	0.68	−0.41	−0.24	0.38
	Kendall’s tau	−0.34	0.20	0.13	−0.10	−0.13	0.10
	Var(S)	1095.67	1095.67	1095.67	1095.67	1095.67	1095.67
	alpha	0.05	0.05	0.05	0.05	0.05	0.05
	*p* value	0.03	0.23	0.41	0.57	0.43	0.54
Humidex	Mann–Kendall test (z,s)	1.56,52.0	2.01,67.0	1.91,64.0	1.18,40.0	0.97,33.0	1.6,54.0
	Sen’s slope estimator	0.02	0.02	0.04	0.02	0.03	0.04
	Kendall’s tau	0.26	0.33	0.31	0.19	0.16	0.26
	Var(S)	1069.33	1077.67	1088.00	1088.00	1089.00	1090.67
	alpha	0.05	0.05	0.05	0.05	0.05	0.05
	p value	0.12	0.04	0.05	0.24	0.33	0.11

Alpha: The significant level.

*p*-value: The estimated probability.

Climate change has affected the weather in Thailand. Table 3 shows the max, min, mean and standard deviation for ambient temperature, relative humidity and precipitation during a 20-year period by meteorological region. The highest average temperatures were recorded in the eastern and central regions (28.16 °C), and the northern region (25.8 °C) presented the lowest average temperature. The southern region had the highest relative humidity at 81.10%, while the northeast region had the lowest (73.48%). Gavrilov et al. (2020) suggested that the aridity index can be calculated for different time scales and is widely utilized worldwide to determine dry or humid climate conditions in various regions. Figure 2 shows the seasonal distribution of the regions, in which 3 regions (central, eastern and southern) had positive humidity values and the remaining regions (northern, northeast and western) had negative humidity values. The HD was calculated for each station based on related maximum temperatures and hygrometric data. The results revealed that the highest average HD values in the eastern, southern and central regions were greater than 38, but the western and northeast regions had values of 36. The lowest value was 34, which was recorded in the northern region. According to the findings in Table 3, all regions of Thailand reported “some discomfort” and “great discomfort or avoid exhaustion”. Among the regions, high temperature and relative humidity in some years with a maximum HD higher than 40 were found only in the southern region, where people experienced considerable discomfort^42,43^. These environments are affected by tropical high pressure, which begins earlier in the southern region and decreases later in the other regions of Thailand, preventing the increase in heat and humidity that is overtly saturated in warmer months of the year, causing heat stress.

The Mann–Kendall (MK) test for simple nonparametric data was employed to study the slopes to show the output of the trend, and Sen’s slope estimator was evaluated using MAKESENS^14^. The positive sign represented an increasing slope, and the negative sign represented a decreasing slope; a zero slope represented no trend in the data for the study period^1^. Sen’s slope estimates are shown in Table 4 and Figs. S1–S2 for the annual temperature, relative humidity, and precipitation for the six regions of Thailand between 2001 and 2020. All six regions showed upward trends, and Sen’s slope estimates determined by the MK statistic (Z) had positive values of 1.58, 0.56, 0.98, 0.73, 0.97, and 1.80. However, the upward trend was not significant at the 5% significance level since the calculated p value was greater than the significance level. Thus, the ITA method was applied to the annual temperature data between 2001 and 2020. In addition, the graph showing the distance of the data points lying on the 1:1 line indicated no trend in the data. If the data point is displayed over a triangle, it indicates that the trend is an upward trend. In contrast, the data points that lie under the triangle represent a downward trend.

The findings of the ITA method for the six regions (northern, southern, eastern, western, northeast, and central regions) are presented in the supplementary data (Fig. S1). The temperature results showed that most data points fell above the 1:1 line, implying an overall upward trend, and this finding agreed strongly with the positive MK test values. However, the upward trend was not significant at the 5% significance level since the calculated p value was greater than the alpha level (0.05).

Relative humidity in the northern, western and central regions was found to have a decreasing slope, and the MK statistic (Z) had a negative value. The western region had a significant upward trend at the 5% significance level since the calculated p value was less than the alpha level (0.05). On the other hand, the southern, eastern, and northeast regions had upward trends, but these were not significant at the 5% significance level since the calculated p value was greater than the alpha level.

Furthermore, the ITA showed that there was a tendency towards abrupt pattern changes in the time series data in three regions, and they had the same trend features; relative humidity below 75% showed no trend. If the relative humidity greater than 75% had shown an upward trend, it would have agreed with the positive MK test values. However, the upward trend was not significant at the 5% significance level since the calculated p value was greater than the alpha level (0.05). In the annual time series data, precipitation in the northern and northeastern regions showed a downward trend, and this finding agreed with the Z statistic in the MK test. There was a significant decrease in the trend at the 5% significance level since the computed p value was less than the alpha level (0.05). Contradictorily, the other 4 regions (southern, eastern, western, and central regions) showed positive trends. The MK test (Z) implied an upward trend over time, as the southern, western and central regions showed positive trends, but the trend was not significant at the 5% significance level because the calculated p value was greater than the alpha level (0.05).

Fig. S2 shows the distribution of annual precipitation using the ITA method. The results demonstrated that the precipitation trends in the six regions had differences and similarities. Low-intensity precipitation showed a downward trend in 4 regions, while high-intensity precipitation showed an upward trend. In contrast, the southern and eastern regions were recorded as having high precipitation and had a significant upward trend. In particular, the increase in precipitation was significantly greater than that in the northern region, but the trend was not significant at the 5% significance level because the calculated p value was greater than the alpha level (0.05) in the MK test.

In this study, annual humidex data between 2001 and 2020 for the six regions of Thailand were used to calculate the trend using the MK test. In Table 4, the results of the Z statistic in the MK test for the humidex show upward trends in all regions. Sen’s slope estimator determined that the MK statistic (Z) was positive at the 0.50 significance level and indicated a significant upward trend. All regions showed an upward trend, with the calculated Z values per year being between 0.97 and 2.01. The ITA method has been applied in studies using the humidex. In this study, the ITA method revealed that most data points fell above the 1:1 line, implying an overall upward trend. The southern and eastern regions showed a significant upward trend at the 5% significance level since the calculated p value was lower than the alpha level (0.05), according to the MK test.

The results from the MK test and ITA were in line with findings from other studies in the literature. Analysis of other studies in the field revealed a consistency between the findings derived from the MK test and ITA. An investigation of air temperature and precipitation patterns in China and Turkey utilized linear regression analysis, Sen's slope estimator, the MK test, and the ITA method. The authors underscored the usefulness of the ITA method and did not make assumptions in determining trends in hydrometeorological parameters^20,44^.

The analysis of extreme climate indicators has been carried out in various areas worldwide, including America, Africa, Europe, and Asia. Data have shown that relative humidity and precipitation have decreased, in contrast with Jakarta, between 1866 and 2010^45^, whereas temperature and the humidex have been on the uptrend according to studies in China^44^. Therefore, it can be concluded that the observation of different trend situations across different periods can lead to changes in trends over time, highlighting the importance of investigating partial trends.

The finding from this study might benefit to understanding the climate changing pattern throughout the country, which could potentially apply to setting the planning and policy in terms of agricultural product and tourism management such as open/ close times of national park as well as the public relation for tourist. Moreover, the changing of the climate parameters would be a benefit for the prediction of pathogen that could be a cause of diseases burden in Thailand.

## Conclusion

The annual variations in temperature, relative humidity, precipitation and humidex were analyzed by using the Mann–Kendall test and the ITA method between 2001 and 2020 in six regions of Thailand. The conclusions of our study can be summarized as follows:

The Mann–Kendall (MK) test and innovative trend analysis (ITA) method were used to analyze the trends in annual temperature, relative humidity, precipitation, and humidex data for six regions in Thailand from 2001 to 2020. The MK test results showed that most regions had an upward trend for temperature and humidex, but the trends were not significant at the 5% significance level. However, for relative humidity and precipitation, some regions showed a significant downward or upward trend at the 5% significance level.

The ITA method was also applied to the data, and it provided graphical representations of the trends. The ITA results supported the MK test results for temperature and humidex data, as most data points fell above the 1:1 line, indicating an overall upward trend. For relative humidity and precipitation data, the ITA results showed that the trends varied across the regions.

The findings of this study are helpful for adaptation plan management involved in predicting the risk associated with current climate impacts substantially affected by hydrometeorological variables, namely, temperature, humidity, evaporation and precipitation. Similarly, hydrological events such as floods and droughts have an influence on climate impacts. This study also contributes to trend detection methods by quantitatively evaluating the trends of time series data. Overall, this study provides insights into the climate trends in different regions of Thailand and highlights the importance of using both statistical and graphical methods to analyze trends in environmental data. Due to the lack of data, this study analyzed annual trends in the data in only six regions. Future studies can collect a greater amount of data to analyze monthly and seasonal changes.

### Supplementary Information

Supplementary Information.

## Data Availability

The data used and analyzed in the manuscript will be made available upon request to the corresponding author.
